# DNER promotes epithelial–mesenchymal transition and prevents chemosensitivity through the Wnt/β-catenin pathway in breast cancer

**DOI:** 10.1038/s41419-020-02903-1

**Published:** 2020-08-18

**Authors:** Zhong Wang, Zhiyu Li, Qi Wu, Chenyuan Li, Juanjuan Li, Yimin Zhang, Changhua Wang, Si Sun, Shengrong Sun

**Affiliations:** 1 Department of Breast and Thyroid Surgery, Renmin Hospital of Wuhan University, Wuhan, Hubei China; 2 Department of Pathophysiology, Wuhan University School of Basic Medical Sciences, Wuhan, Hubei China; 3 Department of Clinical Laboratory, Renmin Hospital of Wuhan University, Wuhan, Hubei China

**Keywords:** Breast cancer, Chemotherapy, Oncogenes

## Abstract

Breast cancer (BC) is the most common malignant tumour in women worldwide, and one of the most common fatal tumours in women. Delta/Notch-like epidermal growth factor (EGF)-related receptor (DNER) is a transmembrane protein involved in the development of tumours. The role and potential mechanism of DNER in epithelial–mesenchymal transition (EMT) and apoptosis in BC are not fully understood. We find that DNER is overexpressed in BC tissue, especially triple-negative breast cancer (TNBC) tissue, and related to the survival of BC and TNBC patients. In addition, DNER regulates cell EMT to enhance the proliferation and metastasis of BC cells via the Wnt/β-catenin pathway in vitro and in vivo. Moreover, the expression levels of β-catenin and DNER in BD tissue are positively correlated. The simultaneously high expression of DNER and β-catenin contributes to poor prognosis in BC patients. Finally, DNER protects BC cells from epirubicin-induced growth inhibition and apoptosis via the Wnt/β-catenin pathway. In conclusion, these results suggest that DNER induces EMT and prevents apoptosis by the Wnt/β-catenin pathway, ultimately promoting the malignant progression of BC. In conclusion, our study demonstrates that DNER functions as an oncogene and potentially valuable therapeutic target for BC.

## Introduction

Breast cancer (BC) is the most common malignant tumour in women worldwide and one of the most common fatal tumours in women^1,2^. BC treatments can be used to improve patient outcome^3^. However, tumour recurrence and metastasis and chemotherapeutic resistance are the most common causes of cancer treatment failure. Therefore, the need to screen and identify key regulatory factors in the process of tumour recurrence and metastasis for the treatment of BC is urgent.

Tumour EMT is a multifactorial and complex event in which epithelial properties and the ability to adhere to adjacent cells are lost and mesenchymal and stem cell phenotypes are eventually obtained^4–7^. EMT, a crucial regulatory mechanism by which tumours acquire invasive and metastatic abilities and the ability to resist apoptosis, plays an irreplaceable role in the development of malignant tumours^8–10^. Recent studies upon activation of the classical Wnt/β-catenin pathway, β-catenin enters and accumulates in the nucleus, which induces the transcription and translation of downstream target genes, thus accelerating EMT^10^. Therefore, maintaining β-catenin activity is important for the Wnt/β-catenin pathway and tumour progression.

DNER, a neuron-specific transmembrane protein found in a variety of peripheral cells^11–14^, is a member of the atypical Notch ligand family and binds to Notch1 receptor^11,15^. DNER is expressed at abnormally high levels in various cancer tissues^16^ and promotes the proliferation migration, and invasion of cancer cells^16,17^ but has an inhibitory effect on cell proliferation in glioma^14^. Nevertheless, the precise function and underlying molecular mechanisms of EMT and chemosensitivity in BC are unclear.

In this study, we have revealed the previously unrecognized role of DNER in cancer progression, EMT and the apoptosis of BC cells. Furthermore, we investigated the expression of DNER and its relationship with survival in BC and TNBC patients. In addition, we have provided evidence for the correlation between DNER and β-catenin and the prognostic value of the high-level expression of DNER and catenin in BC patients. Finally, the crucial role of β-catenin in DNER-induced EMT and the inhibitory effect of DNER on apoptosis have been revealed. Taken together, our results elucidate the potential functions and mechanism of DNER in EMT and apoptosis in BC cells and provide a new therapeutic pathway for the recurrence, metastasis and chemotherapy resistance of BC.

## Materials and methods

### Ethics statement

Two groups of the same human tissue specimens were acquired from patients of Renmin Hospital of Wuhan University who were diagnosed with BC from 2010 to 2012. One group of specimens was promptly stored at −80 °C for western blotting and PCR analysis. The other group of specimens was fixed in formalin and paraffinized for immunohistochemistry (IHC). All patients did not receive chemotherapy, radiotherapy or immunotherapy. This research was approved by the Ethics Committee of Renmin Hospital of Wuhan University, and informed consent was obtained from all patients.

### Cell culture and reagents

Human BC cell lines (MCF-7 and MDA-MB-468 cells) were obtained from American Type Culture Collection and incubated by their corresponding recommended method. All cell lines were mycoplasma-free by morphological examination and verified for their authenticities by STR profiling. Epirubicin was purchased from Pfizer Pharmaceutical Co., Ltd. (Wuxi, China) and dissolved in physiological saline. CHIR 99021 (β-catenin inhibitor) and XAV-939 (β-catenin agonist) were purchased from Selleck (Shanghai, China) and dissolved in DMSO.

### Immunohistochemical staining

IHC staining was performed as previously described^18^. The results of IHC staining were evaluated by two independent pathologists and scored according to the percentage of positive tumour cells and staining intensity. The percentage of positive cells was scored as follows: 0 < 10%, 1 = 10–20%, 2 = 21–50% and 3 > 50%. The staining intensity was evaluated as follows: 0 = no staining, 1 = weak staining, 2 =;moderate staining and 3 =;strong staining. The final protein staining score was the percentage score multiplied by the intensity score; final protein staining scores were divided into three categories as follows: 0 = negative, 1–3 = low expression and 4–9 = high expression.

### siRNA and plasmid transfection

DNER siRNA (5′-GCUUUGCCAGUCCAAGAUUTT) and scramble siRNA (5′-UUCUCCGAACGUGUCACGUTT) were synthesized from GenePharma Co. (Shanghai, China). FLAG-DNER and FLAG-NC were purchased from GeneChem Co. (Shanghai, China). When cells in a six-well plate had grown to the appropriate density, siRNA and plasmids were transiently transfected with Lipofectamine3000 (Invitrogen, USA) and RNAiMAX (Invitrogen, USA), respectively, according to the manufacturer’s instructions. After 48 h of transfection, the cells were used for subsequent experiments.

### qRT-PCR

Total RNA from tissue specimens and cell samples was extracted by using TRIzol (Invitrogen, USA) according to the protocol and then reverse transcribed to cDNA using a TransScript First-Stand cDNA Synthesis Kit (TaKaRa, Japan). qRT-PCR was implemented by using SYBR Green Mastermix (TaKaRa, Japan) with an ABI 7900HT Real-Time PCR system (USA). The primer sequences are shown in Supplemental Table 1.

### Cell Counting Kit (CCK)-8 assay

After a series of interventions, equal numbers of BC cells were plated into 96-well plates and cultured for 4 days. Ten microlitres of CCK-8 (CK04, Dojindo, Japan) solution was added to each well, and the cells were incubated at 37 °C for 2 h. The absorbance was determined at 450 nm.

### Wound healing assay

After intervention, the cells were seeded into six-well plates. When the cell density exceeded 90%, the cells were washed twice with PBS, and scratches were made with a yellow plastic pipette tip. Cells were cultured in serum-free medium for 48 h and photographed under a microscope.

### Invasion assay

After a series of treatments, 4 × 10^4^ cells in serum-free medium were plated in the upper chambers of a Transwell apparatus with Matrigel (Corning, NY, USA). Medium in the bottom chambers containing 10% FBS served as an attractant. After 24 h of incubation, cells that passed through the chamber membrane were fixed with pre-cooled formaldehyde and stained with crystal violet (C0121, Beyotime). The cells were counted and photographed under a microscope.

### Western blotting

The prepared tissue and cell samples were separated by protein SDS-PAGE and transferred to a nitrocellulose (NC) membrane. The membrane was blocked in 5% skim milk powder for 1.5 h at room temperature and immunoblotted with primary antibody at 4 °C overnight. After incubation with secondary antibody at room temperature for 1 h, protein expression was detected with corresponding protein development instrument and quantified by ImageJ software (W S Rasband, Image J, NIH). The antibodies used are listed in Supplementary Table 2.

### Nuclear and cytoplasmic protein extraction

Nuclear and Cytoplasmic Extraction Reagent (P0027) was purchased Beyotime Biotechnology. The nuclear and cytoplasmic proteins were extracted according to the instructions and then used for subsequent experiments.

### Flow cytometry to detect apoptosis

A FITC Annexin V Apoptosis Detection Kit I (556547, BD Pharmingen, USA) was used to detect cell apoptosis. The cells were seeded in six-well plates. After a series of interventions, cells were processed following the manufacturer’s protocol, and the cell fluorescence was measured with a FACScan flow cytometer (FACScan, Becton Dickinson).

### Animal experiments

To acquire MDA-MB-468 cells with DNER stably knocked down and MCF-7 cells stably overexpressing DNER, cells were transfected with DNER knockdown and overexpression lentivirus (GeneChem, Shanghai, China) and then selected with puromycin. When the transfection efficiency approached ~90%, the DNER protein level was detected with western blotting. All experimental procedures were conducted according to the Regulations of Experimental Animal Administration issued by the Animal Committee of Wuhan University. The mice were randomly divided into two groups. A total of 4 × 10^6^ stable cells in 250 μl PBS were subcutaneously inoculated into the right iliac fossa of 4- to 5-week-old female athymic nude mice (BALB/c). After a certain period of intervention, the mice were sacrificed by anaesthesia, and xenografts were removed for weighing and photographing. The expression of relative proteins was detected by western blotting and IHC.

For mammary-fat-pad tumour assays, we established MDA-MB-231 cells with DNER stably knocked down. The mice were randomly divided into two groups. 1.5 × 10^6^ stable cells were resuspended in a mixture of PBS and Matrigel (1:1) and then injected into the fourth mammary fat pad on the same side of nude mice. To observe lung metastasis, tumours were excised by surgical operation when they reached about 300 mm^3^. Ten days after the operation, the mice were sacrificed by anaesthesia, and the number of metastatic tumours per lung were determined. The entire lung tissues were fixed with 10% formalin and sectioned for haematoxylin and eosin (H&E) staining to determine the presence of lung metastasis. The entire lung tissues were fixed with 10% formalin and sectioned for haematoxylin and eosin (H&E) staining to determine the presence of lung metastasis.

### Immunofluorescence

Immunofluorescence staining was performed as previously described^19^. In brief, after corresponding treatments, the cells fixed with 4% paraformaldehyde were perforated by 0.2% Triton-X 100 for 15 min and blocked with 5% BSA for 1 h. Next, the cells were incubated with β-catenin (1:100 dilution) overnight at 4 °C and then incubated for 30 min with 488-conjugated antibody (Invitrogen, A11034). Finally, the slides were stained with DAPI for 3 min. The images of sample were analyzed by laser confocal microscopy (Zeiss LSM 710).

### Statistical analysis

Statistical analyses were performed using SPSS 24.0 software (SPSS Inc., Chicago, IL) and GraphPad Prism 8 (GraphPad Software, La Jolla, CA, USA). All data were analyzed with at least three independent experiments and are presented as the mean ± SD. A survival curve was prepared by Kaplan–Meier analysis, and the log-rank test was used to compare survival differences between groups. Pearson’s correlation method was used to analyze the correlation between DNER and β-catenin. A chi-square test was used to analyze associations between DNER expression levels and clinical characteristics. One-way ANOVA was used to compare differences in three or more groups. Differences in which *p* < 0.05 were considered statistically significant.

## Results

### DNER is upregulated in BC tissues and correlated with poor prognosis in BC and TNBC patients

**Fig. 1:**
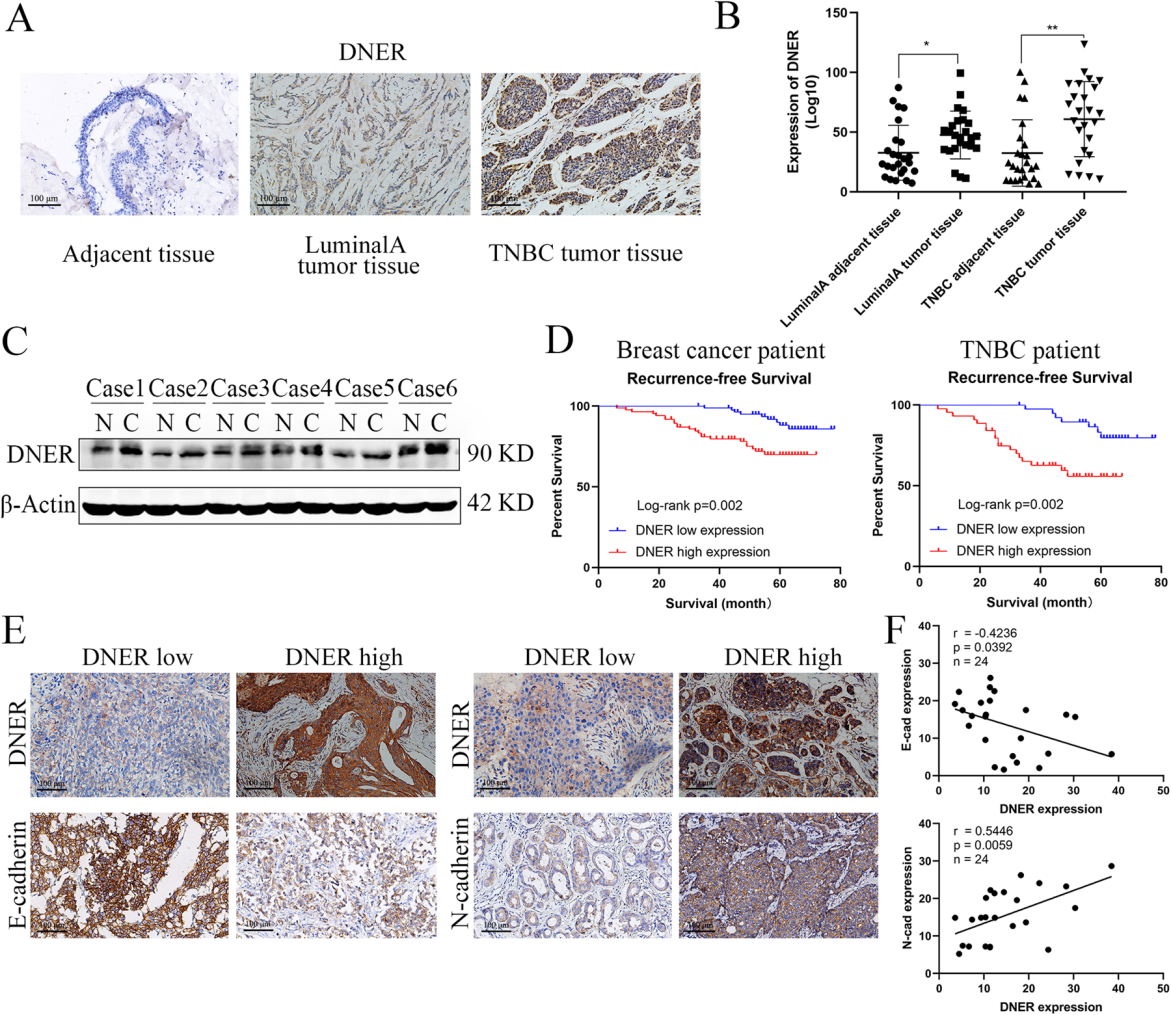
DNER is upregulated in BC tissues and correlated with poor prognosis in BC and TNBC patients. **a** The expression levels of DNER in luminal A and TNBC tumour tissues compared with adjacent tissue by IHC (magnification ×200). **b** The mRNA levels of DNER in luminal A and TNBC tumour tissues compared with adjacent tissue. **c** The DNER protein expression in BC tissues and adjacent tissues by western blotting. **d** The Kaplan–Meier analysis showed the RFS of BC and TNBC patients with DNER high expression or DNER low expression. **e** The staining of DNER, E-cadherin and N-cadherin in BC tissue by IHC (magnification ×200). **f** Correlation analyses of protein expression levels between E-cadherin, N-cadherin and DNER. *p < 0.05, **p < 0.01 vs the control group.

To determine the role of DNER in development of BC, we first measured the expression levels of DNER in BC tissue and matched adjacent normal breast tissue by IHC. The expression level of DNER in BC tissue was markedly higher than that in adjacent tissue; moreover, the expression in TNBC was higher than that in luminal A BC (Fig. 1a). We also detected the expression of DNER in BC tissue by PCR, the results of which were consistent with those of IHC experiments (Fig. 1b). To further verify DNER expression in BC, we utilized western blotting to detect DNER protein expression in BC and adjacent tissues. As expected, compared with DNER expression in adjacent tissues, DNER expression in BC tissues was significantly elevated (Fig. 1c). Furthermore, the highest DNER expression level was found in TNBC tissue. The clinicopathological characteristics with different expression of DNER in all BC and TNBC patients were shown in Tables 1 and 2. Kaplan–Meier analysis of RFS showed that the group expressing high levels of DNER had a worse prognosis than the group expressing low levels of DNER. The results of survival analysis of TNBC patients were the same as that of BC patients, and TNBC patients had a shorter RFS than BC patients (Fig. 1d). Next, to verify whether the poor prognosis of BC patients caused by DNER is related to EMT, we detected the correlation between DNER- and EMT-related markers. The results showed that DNER expression was negatively correlated with the expression of E-cadherin, while positively correlated with N-cadherin expression (Fig. 1e, f). In addition, we found that high expression of mesenchymal markers was significantly associated with high expression of DNER in BC through the TCGA database (http://gepia.cancer-pku.cn/). Although the negative correlation between E-cadherin and DNER in TCGA database was not significant, it also presented a negative trend (Supplementary Fig. 2A). The results therefore suggested that DNER is highly expressed in BC and that elevated DNER protein expression contributes to the progression of BC, especially TNBC.

**Table 1:** Clinicopathological associations of DNER expression in breast cancer.

Variables	Low *N* = 92 (%)	High *N* = 114 (%)	*P* value^*^
Age at diagnosis, years			0.664
≤50	21 (22.8)	29 (25.4)	
>50	71 (77.2)	85 (74.6)	
Grade			**0.043**
Well	17 (18.5)	9 (7.9)	
Moderately	24 (26.1)	26 (22.8)	
Poorly	51 (55.4)	79 (69.3)	
Tumour size (cm)			0.073
≤2	47 (51.1)	44 (38.6)	
>2	45 (48.9)	70 (61.4)	
Lymph node metastasis			0.759
Negative	35 (38.0)	41 (36.0)	
Positive	57 (62.0)	73 (64.0)	
Vascular invasion			0.098
Negative	78 (84.8)	86 (75.4)	
Positive	14 (15.2)	28 (24.6)	
ER			0.152
Negative	69 (75.0)	75 (65.8)	
Positive	23 (25.0)	39 (34.2)	
PR			**0.034**
Negative	55 (59.8)	84 (73.7)	
Positive	37 (40.2)	30 (26.3)	
HER2			0.381
Negative	58 (63.0)	65 (57.0)	
Positive	34 (37.0)	49 (43.0)	
Ki67			0.535
<14 %	72 (78.3)	85 (74.6)	
≥14 %	20 (21.7)	29 (25.4)	
Recurrence			**0.041**
No	83(90.2)	91(79.8)	
Yes	9(9.8)	23(20.2)	

*P values calculated by log-rank testing; bold if statistically significant, *P* < 0.05.

*ER* oestrogen receptor, *PR* progesterone receptor, *HER2* human epithelial growth factor receptor-2.

**Table 2:** Clinicopathological associations of DNER expression in triple negative breast cancer.

Variables	Low *N* = 47 (%)	High *N* = 51 (%)	*P* value^*^
Age at diagnosis, years			0.818
≤50	12 (25.5)	12 (23.5)	
>50	35 (74.5)	39 (76.5)	
Grade			**0.037**
Well	6 (12.8)	2 (3.9)	
Moderately	12 (25.5)	6 (11.8)	
Poorly	29 (61.7)	43 (84.3)	
Tumour size (cm)			**0.048**
≤2	23 (48.9)	15 (29.4)	
>2	24 (51.1)	36 (70.6)	
Lymph node metastasis			0.113
Negative	25 (53.2)	19 (37.3)	
Positive	22 (46.8)	32 (62.7)	
Vascular invasion			0.398
Negative	35 (74.5)	33 (64.7)	
Positive	12 (25.5)	18 (35.3)	
Ki67			0.481
<14 %	30 (63.8)	29 (56.9)	
≥14 %	17 (36.2)	22 (43.1)	
Recurrence			**0.021**
No	40 (85.1)	33 (64.7)	
Yes	7 (14.9)	18 (35.3)	

^*^P values calculated by log-rank testing; bold if statistically significant, *P* < 0.05.

### DNER increases the biological functions of BC cells in vitro

**Fig. 2:**
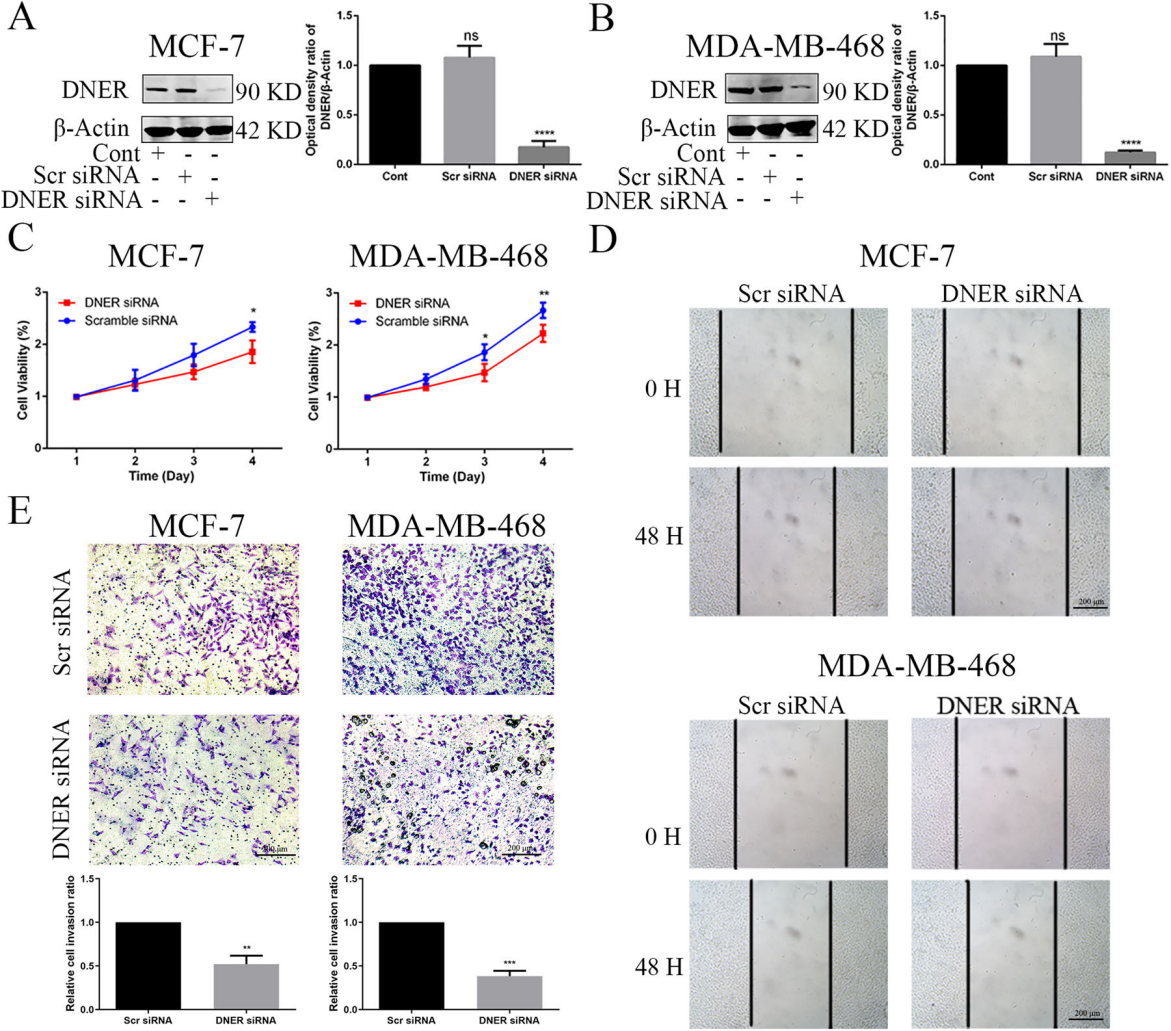
DNER knockdown inhibits cell proliferation and metastasis of BC cells. **a**, **b** The knockdown efficiency of DNER in MCF-7 and MDA-MB-468 cells. **c** Cell growth was measured by CCK-8 assay after DNER knockdown in two BC cell lines. **d** Wound healing assay was used to determine the migratory ability of BC cells with DNER knockdown. **e** The invasion capacity of BC cells with knockdown of DNER was confirmed by Transwell assay. Down: Quantitative analysis of invasion ratio was shown. The values are the mean ± SD from three independent experiments. ^ns^*p* > 0.05, *p < 0.05, **p < 0.01, ***p < 0.001, ****p < 0.0001 vs the control group.

To evaluate the effect of DNER on BC cell proliferation, migration and invasion, we used siRNA to suppress DNER expression in both MCF-7 and MDA-MB-468 cells. Compared with DNER expression in the control and scramble siRNA groups, DNER was silenced by almost 90% and 83% in MCF-7 and MDA-MB-468 cells transfected with siRNA, respectively, (Fig. 2a, b). As shown in Fig. 2c, DNER knockdown visibly downregulated the growth rate of BC cells by CCK-8 assay. Next, a wound healing assay was used to evaluate cell migration capacity. Compared with wound closure in the scramble siRNA group, DNER knockdown significantly inhibited wound closure after 48 h in BC cells (Fig. 2d). In addition, the Transwell assay revealed that DNER knockdown clearly reduced BC cell invasion (Fig. 2e). These results suggest that DNER acts as a cancer-promoting gene in BC cells.

To further confirm the role of DNER in BC progression, DNER was overexpressed by transfection with the FLAG-DNER plasmid for 48 h. As shown in Supplementary Fig. 1A, DNER was successfully overexpressed in the two BC cell lines. In striking contrast with the effects of DNER knockdown, the ability of cell proliferation, migration and invasion was markedly enhanced after DNER overexpression (Supplementary Fig. 1B–E). Taken together, these results indicated that DNER plays a crucial role in BC growth and metastatic potential.

### DNER induces EMT in BC cells

**Fig. 3:**
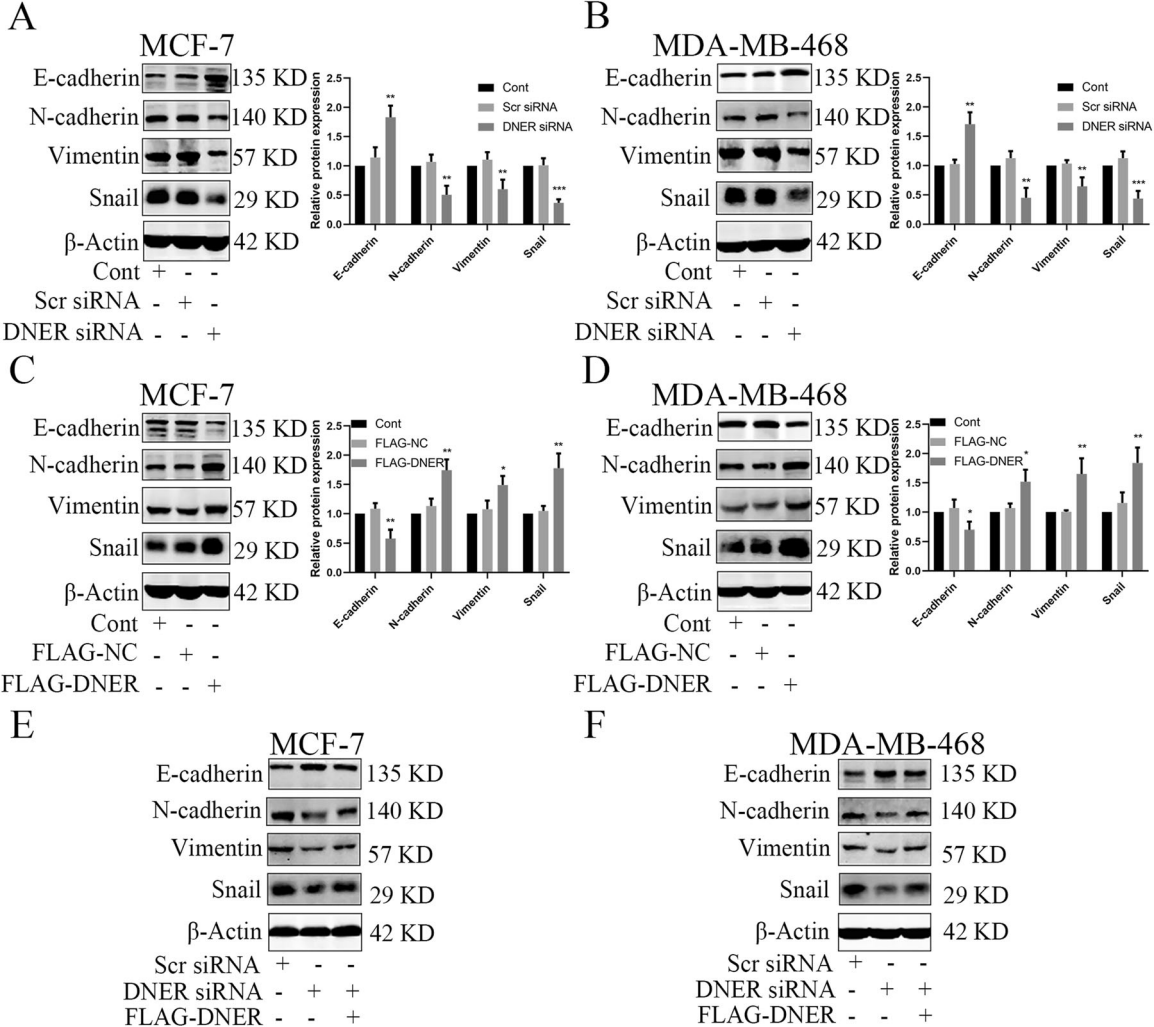
DNER induces EMT in BC cells. **a**, **b** EMT-related proteins E-cadherin, N-cadherin, Vimentin, and Snail were detected by western blotting in DNER knockdown cells. Right: quantitative analysis of the optical density ratio of E-cadherin, N-cadherin, Vimentin and Snail compared with β-actin are shown. **c**, **d** EMT-related protein levels were measured by western blotting after DNER overexpression in BC cells. Right: quantitative analysis of the optical density ratio of E-cadherin, N-cadherin, Vimentin, and Snail compared with β-actin are shown. **e**, **f** DNER was overexpressed in DNER knockdown cells, and then western blotting detected the expression of EMT-related proteins. The values are the mean ± SD from three independent experiments. *p < 0.05, **p < 0.01, ***p < 0.001 vs the corresponding group.

Tumour cell EMT promotes the malignant progression and metastasis of tumour cells^10^. We next examined whether DNER has a regulatory effect on BC cell EMT. To assess this function, we detected EMT-related protein expression by western blotting. DNER knockdown significantly upregulated epithelial-like marker (E-cadherin) expression and downregulated mesenchymal marker (N-cadherin, Vimentin, Snail) expression (Fig. 3a, b). Conversely, overexpression of DNER dramatically shown the opposite effect (Fig. 3c, d). These results indicate that DNER drives EMT in BC cells. To provide further evidence of this effect of DNER on EMT, we suppressed DNER expression and then transfected cells with the FLAG-DNER plasmid to restore the DNER protein level; we then determined whether DNER overexpression could reverse changes in the expression of EMT-related proteins. As shown in Fig. 3e, f, DNER knockdown alone had an inhibitory effect on EMT, whereas DNER knockdown and FLAG-DNER transfection suppressed the effect of DNER knockdown on E-cadherin and partially restored the expression of N-cadherin, Vimentin and Snail. These results suggest that DNER plays a pivotal role in inducing EMT in BC cells.

### DNER activates the Wnt/β-catenin signalling pathway and is positively correlated with β-catenin

**Fig. 4:**
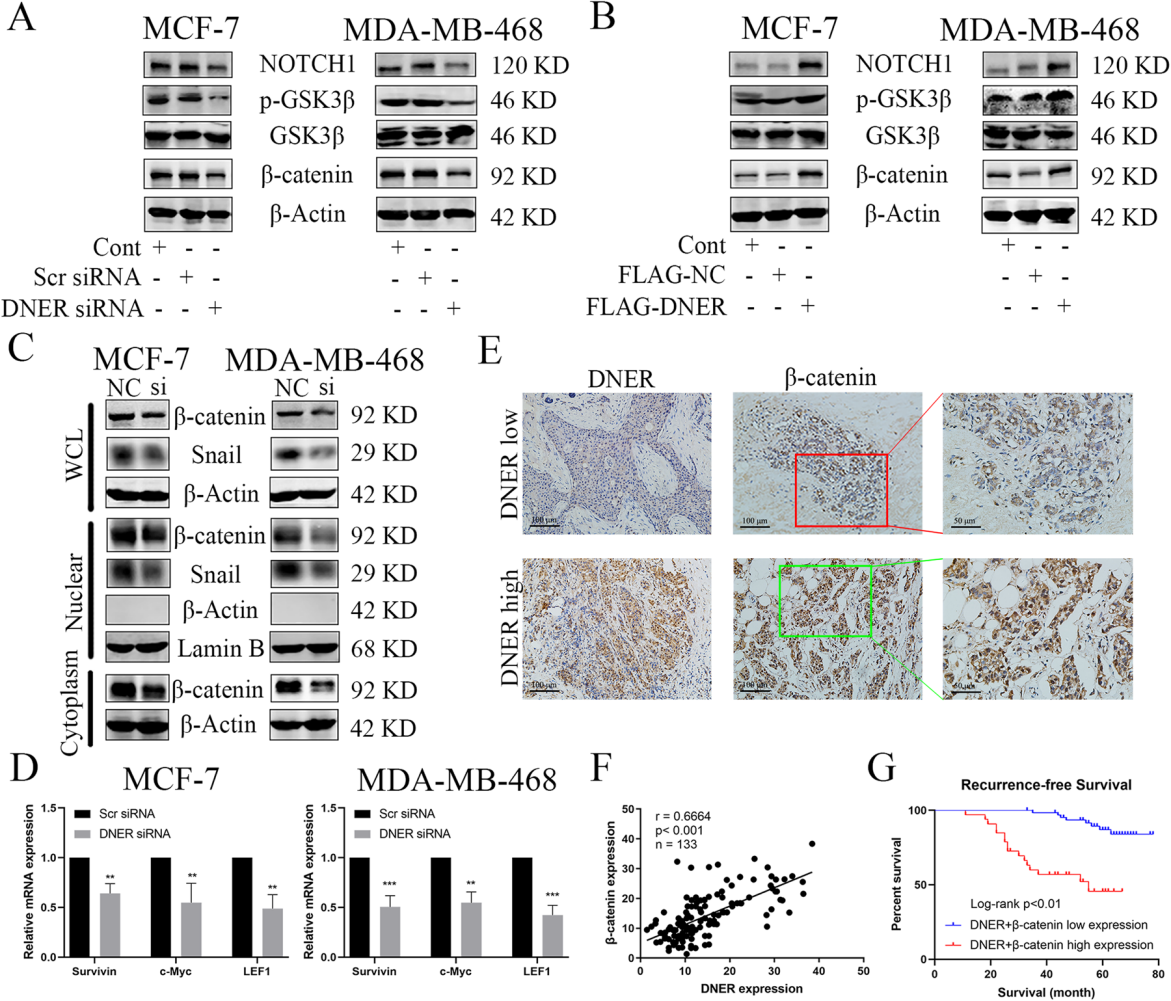
DNER activates the Wnt/β-catenin signalling pathway and is positively correlated with β-catenin. **a**, **b** Western blotting detected the expression of Notch1, p-GSK3β, GSK3β and β-catenin after DNER knockdown or DNER-overexpressing in BC cells. **c**. Total proteins (β-catenin and Snail) nuclear proteins (β-catenin and Snail) in DNER knockdown cells were assayed with western blotting. **d** The mRNA levels of Survivin, c-Myc and LEF1 were detected by qRT-PCR. **e** The staining of DNER and β-catenin in BC tissue by IHC (magnification ×200). **f** Correlation analyses of protein expression levels between DNER and β-catenin. **g** Kaplan–Meier survival analysis of BC patients was performed with DNER^High^β-catenin^High^ and DNER^Low^β-catenin^Low^ expression. The values are the mean ± SD from three independent experiments. **p < 0.01, ***p < 0.001 vs the corresponding group.

Previous reports have shown that the Wnt/β-catenin signalling pathway plays a crucial role in cancer cell metastasis and EMT^20,21^. Therefore, we examined whether DNER mediates the canonical Wnt/β-catenin signalling pathway. As shown in Fig. 4a, b, compared with control cells, in DNER knockdown cells, the protein levels of Notch1, p-GSK3β and β-catenin were increased, and those of GSK3β were unchanged. Conversely, DNER overexpression dramatically shown the opposite effect. Next, we investigate whether there is a relationship between Notch signal and β-catenin in the case of DNER overexpression. In DNER-overexpressing cells, we knocked down Notch1 and found that β-catenin expression was decreased compared with DNER overexpression alone (Supplementary Fig. 2B). Notch1 functioned as an important role in the Wnt/β-catenin pathway, and the activation of Notch1 was positively related to the nuclear translocation of β-catenin^22^. The accumulation of β-catenin in the nucleus plays an important role in the malignant progression of tumours. We assessed the effect of DNER knockdown on nuclear β-catenin accumulation by western blotting and observed that upon the knockdown of DNER, the levels of nuclear β-catenin and Snail were reduced in BC cell lines (Fig. 4c and Supplementary Fig. 2C). The nuclear location of β-catenin detected by immunofluorescence showed the same results as those determined by western blotting (Supplementary Fig. 2D). To further confirm the decrease in nuclear β-catenin accumulation following DNER knockdown, we examined the expression levels of β-catenin downstream target genes in BC cells by PCR. Consistent with the western blotting results, the mRNA expression levels of Survivin, c-Myc and LEF1 were significantly downregulated upon DNER knockdown (Fig. 4d). These data indicated that DNER knockdown can inhibit nuclear translocation and transcriptional activity of β-catenin, thereby controlling the Wnt/β-catenin signalling pathway.

To verify the relationship between DNER and β-catenin, we measured the protein expression levels of DNER and β-catenin in BC tissues. IHC showed that β-catenin was highly expressed when DNER was overexpressed, while catenin levels were low when DNER was knocked down (Fig. 4e). Interestingly, correlation analyses showed that β-catenin expression was positively correlated with the expression of DNER (Fig. 4f). We also found a strong positive correlation between DNER expression and nuclear β-catenin expression (Supplementary Fig. 2E). Furthermore, immunofluorescence analysis showed that DNER overexpression promoted more nuclear accumulation of β-catenin in BC cells (Supplementary Fig. 2F). Finally, Kaplan–Meier analysis showed that the prognosis of BC patients with high levels of DNER and β-catenin was worse than the prognosis of BC patients with low levels of both DNER and β-catenin (Fig. 4g). In addition, we continued to show the correlation between the high-level expression of both DNER and β-catenin and BC patient clinicopathologic features, as shown in Table 3. These data suggest a strong correlation between the expression of DNER with that of β-catenin and high levels of DNER/β-catenin with poor prognosis in BC.

**Table 3:** Clinicopathological associations of both DNER and β-catenin expression in breast cancer.

Variables	Low *N* = 70	High *N* = 36	*P* value^*^
Age at diagnosis, years			0.945
≤50	19 (27.1)	10 (27.8)	
>50	51 (72.9)	26 (72.2)	
Grade			**0.038**
Well	15 (21.4)	2 (5.6)	
Moderately	19 (27.2)	7 (19.4)	
Poorly	36 (51.4)	27 (75.0)	
Tumour size (cm)			**0.004**
≤2	38 (54.3)	9 (25.0)	
>2	32 (45.7)	27 (75.0)	
Lymph node metastasis			0.415
Negative	27 (38.6)	11 (30.6)	
Positive	43 (61.4)	25 (69.4)	
Vascular invasion			0.113
Negative	58 (82.9)	25 (69.4)	
Positive	12 (27.1)	11 (30.6)	
ER			0.665
Negative	58 (82.9)	31 (86.1)	
Positive	12 (27.1)	5 (13.9)	
PR			**0.003**
Negative	40 (57.1)	31 (86.1)	
Positive	30 (42.9)	5 (13.9)	
HER2			0.057
Negative	46 (65.7)	30 (83.3)	
Positive	24 (34.3)	6 (16.7)	
Ki67			0.183
<14 %	55 (78.6)	24 (66.7)	
≥14 %	15 (21.4)	12 (33.3)	
Recurrence			**< 0.01**
No	62 (88.6)	20 (55.6)	
Yes	8 (11.4)	16 (44.4)	

*P values calculated by log-rank testing; bold if statistically significant, *P* < 0.05.

*ER* oestrogen receptor, *PR* progesterone receptor, *HER2* human epithelial growth factor receptor-2.

### The Wnt/β-catenin signalling pathway is involved in DNER-induced EMT and pro-metastatic phenotypes

**Fig. 5:**
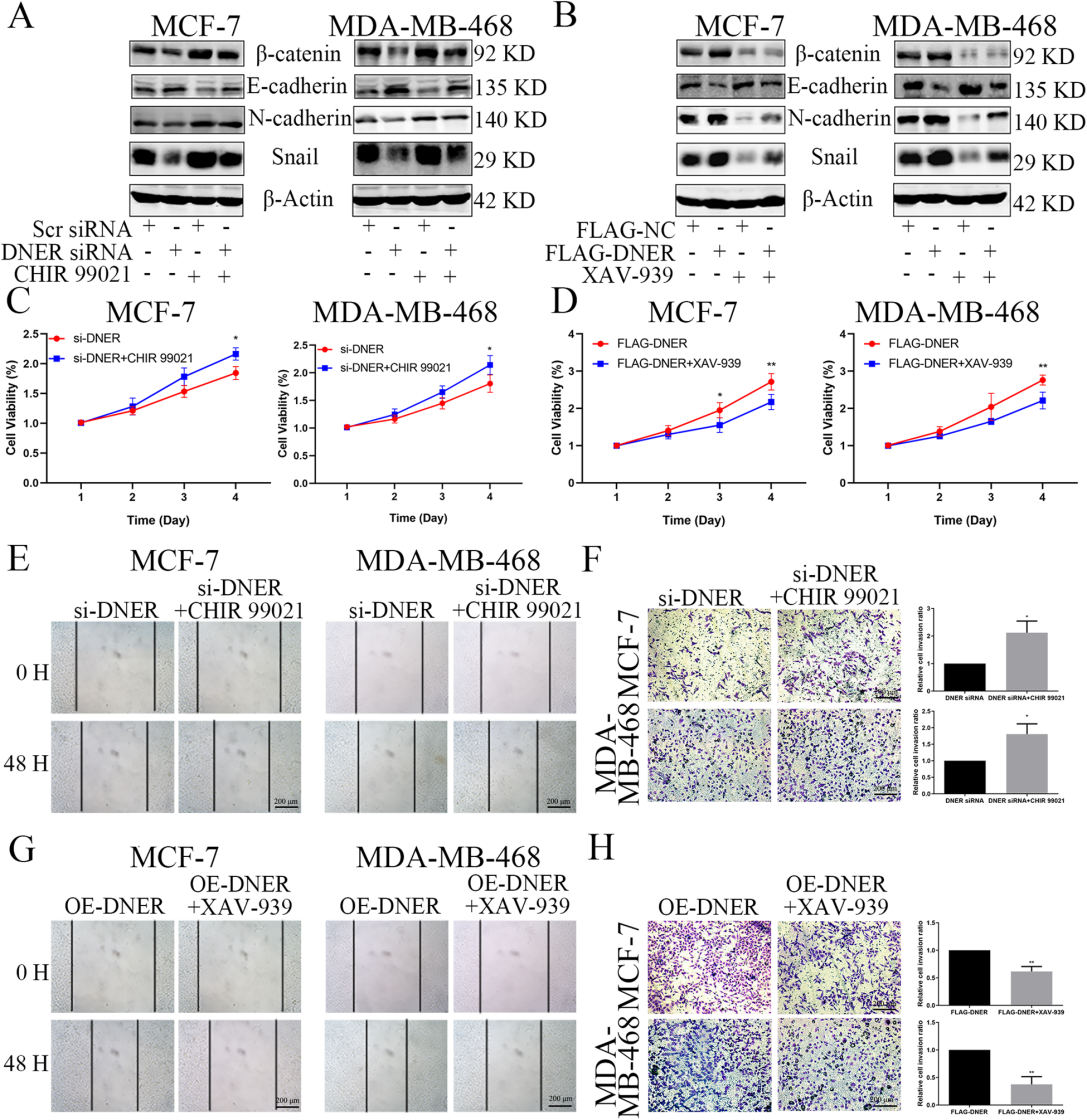
The Wnt/β-catenin signalling pathway is involved in DNER-induced EMT and metastasis. **a**, **b** The expression of EMT-related proteins and β-catenin were detected by western blotting in DNER knockdown or DNER-overexpressing cells with CHIR 99021 (6 μM, 24 h) or XAV-939 (4 μM, 24 h) treatment, respectively. **c**, **d** Cell growth was measured by CCK-8 in BC cells treated as described above. **e**, **g** Wound healing assay was used to examined migration ability in BC cells treated as described above. **f**, **h** Transwell assay showed the cell invasion abilities in BC cells treated as described above. Right: Quantitative analysis of invasion ratio was shown. The values are the mean ± SD from three independent experiments. *p < 0.05, **p < 0.01 vs the corresponding group.

To determine whether the Wnt/β-catenin pathway functions in DNER-induced EMT, we assessed whether CHIR 99021, a specific Wnt/β-catenin pathway activator^23^, and XAV-939, a Wnt/β-catenin pathway inhibitor^24^ could reverse the effect of DNER overexpression and DNER knockdown in BC cells. β-Catenin levels in the two BC cell lines were significantly elevated after CHIR 99021 treatment and markedly suppressed after XAV-939 treatment (Fig. 5a, b). Compared with DNER knockdown alone, levels of the EMT-related proteins were dramatically exhibited the opposite effect after of the treatment of DNER knockdown cells with CHIR 99021 (Fig. 5a). The treatment of DNER-overexpressing cells with XAV-939 clearly show similar results (Fig. 5b). These findings indicated that CHIR 99021 partly rescued the inhibitory effect of DNER knockdown on EMT progression and that XAV-939 suppressed the activation of EMT induced by DNER overexpression. To investigate the role of the Wnt/β-catenin pathway in DNER-mediated cell proliferation, migration and invasion, we performed rescue experiments by activating or inhibiting β-catenin in DNER knockdown or DNER-overexpressing cells, respectively. Consistent with the effects of Wnt/β-catenin pathway activation and inhibition on EMT, in the presence of CHIR 99021, the proliferation, migration and invasion of DNER knockdown cells were clearly elevated (Fig. 5c, e, f). Similarly, inhibition of β-catenin by XAV-939 in DNER-overexpressing cells distinctly decreased metastatic ability, as shown by changes in cell growth, migration and invasion (Fig. 5d, g, h). Altogether, these data suggested that β-catenin is indispensable for DNER-induced BC cell EMT and pro-metastatic phenotypes.

### DNER enhances the tumorigenic and metastatic ability of BC cells in vivo

**Fig. 6:**
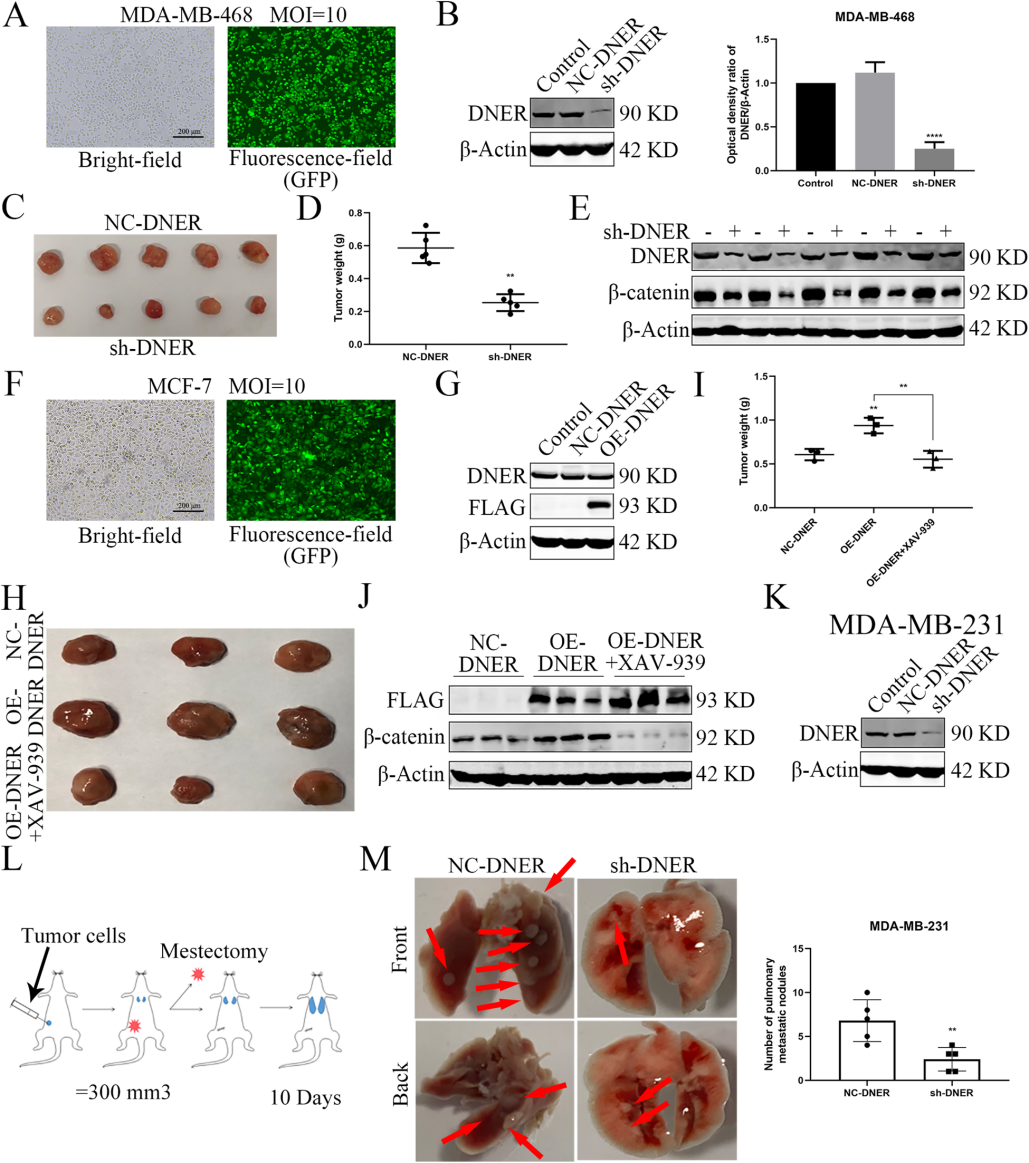
DNER enhances the tumorigenic ability of BC cells in vivo. **a**, **f**, **k** The transfection efficiency of DNER knockdown or expression in MDA-MB-468, MCF-7 or MDA-MB-231 cells, respectively. **b**, **g** The knockdown or overexpression efficiency of DNER in MDA-MB-468 cells or MCF-7 cells, respectively. **c**, **h** The xenograft pictures of sh-DNER and NC-DNER in MDA-MB-468 cells (*n* = 5). **d**, **i** Comparison of tumour weights from various groups. (**e**, **j**) The expression of DNER and β-catenin in xenograft tissue by western blotting. **h** The xenograft pictures of NC-DNER group, OE-DNER group and OE-DNER treated with XAV-939 group in MCF-7 cells (*n* = 3). **l** Schematic diagram of in vivo experimental procedure for lung metastasis potential in situ of BC. **m** Bright imaging of the lungs metastasis (left) and quantification of the metastases tumour (right) generated by MDA-MB231 cells (*n* = 5). **p < 0.01 vs the corresponding group.

To verify our results in vitro, we next examined the role of DNER in vivo. To that end, MDA-MB-468 cells in which DNER was stably knocked down and MCF-7 cells stably overexpressing DNER were successfully established to use to establish xenograft models in mice (Fig. 6a, b, f, g). After a period of time, the xenografts were removed, photographed and weighed. DNER knockdown significantly inhibited tumour size and weight compared with those in NC group (Fig. 6c, d). Consistent with the effect of DNER knockdown, xenografts from DNER-overexpressing group were larger and heavier than those from NC group. More importantly, XAV-939 reversed changes in the size and weight of xenografts (Fig. 6h, i). The DNER, β-catenin, c-Myc and Snail protein levels in xenograft tissue were measured to confirm the upregulation and downregulation by western blotting (Fig. 6e, j, Supplementary Fig. 3A). Moreover, IHC results found that DNER knockdown reduced nuclear location of β-catenin, while DNER overexpression promoted this nuclear translocation effect (Supplementary Fig. 3C). In addition, as shown in Supplementary Fig. 3A, C, the western blotting and IHC results showed that DNER impacted the tumour growth in vivo was related to the level of Ki67, which is consistent with the positive correlation between DNER expression and ki67 expression in BC patients of TCGA database (Supplementary Fig. 3B).

To explore the role of DNER in BC metastasis to lung, MDA-MB-231 cells with stably DNER knockdown was successfully established (Fig. 6k). As shown in Fig. 6l, the corresponding treated MDA-MB-231 cells were injected into the fourth mammary fat pad, and tumours were excised when they reached about 300 mm^3^. Lung metastasis was observed in each group after 10 days. Bright-field picture demonstrated that more lung metastasis was found in the NC-DNER group compared with the sh-DNER group (Fig. 6m). Similar trends were observable in H&E staining analysis (Supplementary Fig. 3E). Moreover, IHC results of xenografts showed that DNER knockdown observably upregulated E-cadherin expression and downregulated N-cadherin expression. DNER overexpression had the opposite effect, whereas XAV-939 reversed the expression levels of EMT markers (Supplementary Fig. 3C, D), indicated that DNER promoted BC metastasis in vivo. These results illuminate that DNER functions as an oncogene in BC.

### DNER reduces the chemosensitivity of BC cells to epirubicin in vitro

**Fig. 7:**
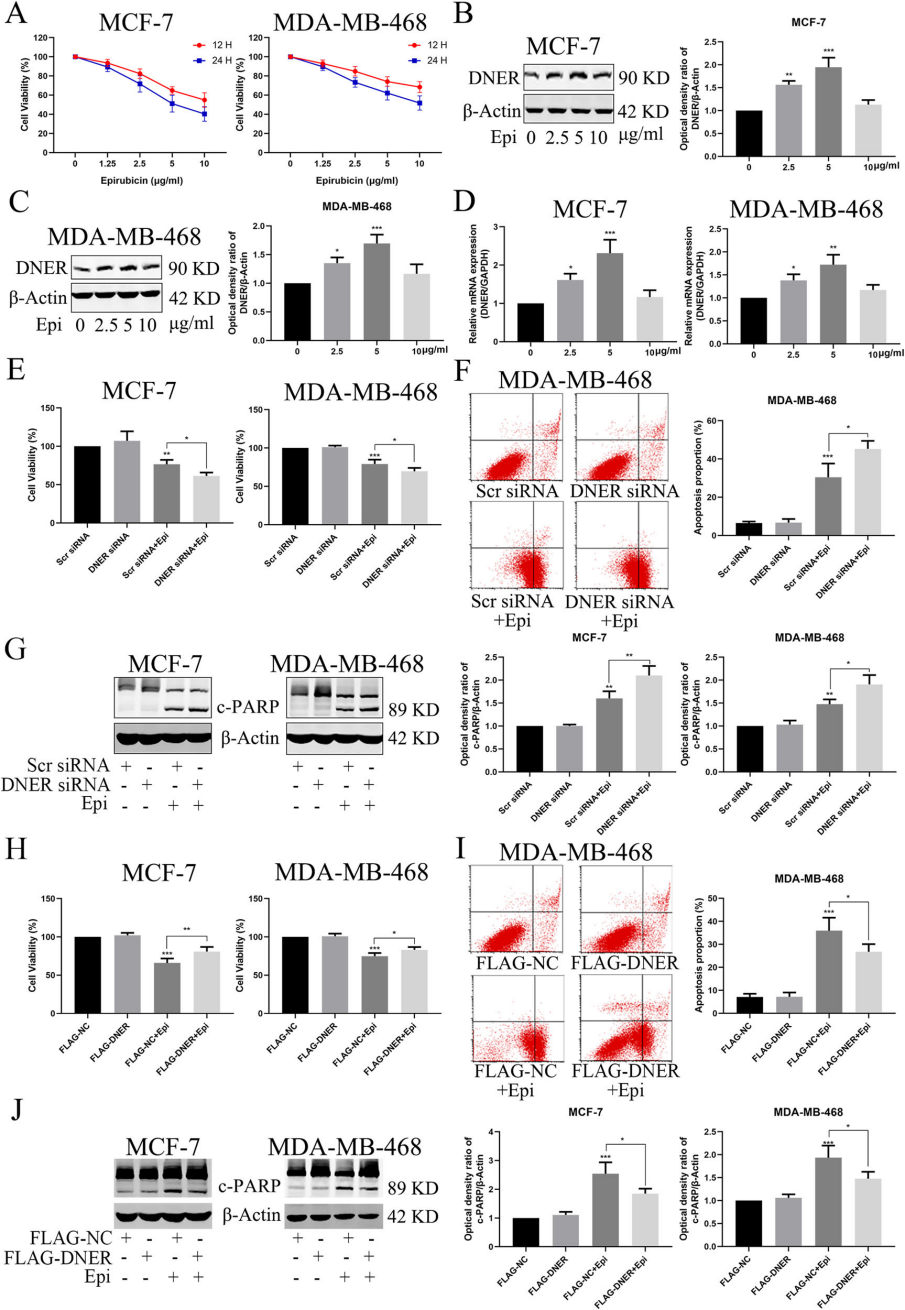
DNER reduces the chemosensitivity of BC cells to epirubicin in vitro. **a** Cell proliferation was detected by CCK-8 after treated with different concentrations of epirubicin in two BC cell lines. **b**, **c** DNER was analyzed by western blotting in BC cells treated as described above. Right: quantitative analysis of the optical density ratio of DNER compared with β-actin are shown. **d** Expression of epirubicin-induced DNER was detected by PCR. **e** Cell viability was assessed by CCK-8 after DNER knockdown treated with epirubicin or not. **f** Analysis of apoptosis with FACS in MDA-MB-468 cells treated as described in (**e**). Right: Quantitative analysis of apoptosis ratio. **g** The expression of PARP was detected by western blotting in BC cells treated as described above. Right: quantitative analysis of the optical density ratio of c-PARP compared with β-actin are shown. **h** Cell growth was measured by CCK-8 after DNER overexpression treated with epirubicin or not. **i** Analysis of apoptosis with FACS in MDA-MB-468 cells treated as described in (**h**). Right: Quantitative analysis of apoptosis ratio. **j** The expression of PARP was detected by western blotting in BC cells treated as described above. Right: quantitative analysis of the optical density ratio of c-PARP compared with β-actin are shown. The values are the mean ± SD from three independent experiments. *p < 0.05, **p < 0.01, ***p < 0.001 vs the corresponding group.

Previous studies have reported that EMT is involved in the chemosensitivity of tumour cells. Our results suggested that DNER promotes EMT in BC cells. Therefore, we examined the relationship between DNER and chemosensitivity to epirubicin. As shown in Fig. 7a, epirubicin inhibited the growth of the two BC cell lines in a concentration- and time-dependent manner. Meanwhile, DNER was elevated at both the protein and mRNA levels after BC cells were treated with different concentrations of epirubicin (1.25–5 μg/ml) for 12 h, but returned to the control level at 10 μg/ml (Fig. 7b–d). Next, we investigated the role of DNER in epirubicin-induced apoptosis. The CCK-8 assay showed that the combination of DNER knockdown and epirubicin treatment had a significant inhibitory effect on cell proliferation compared to that with epirubicin treatment alone (Fig. 7e). Furthermore, DNER knockdown with epirubicin treatment dramatically augmented the apoptosis rate compared with that in the epirubicin group of MDA-MB-468 cells, as determined by fluorescence-activated cell sorting (FACS) (Fig. 7f). The levels of apoptosis-related proteins, such as PARP, detected by western blotting were the same as those determined by FACS (Fig. 7g), indicating the protective role of DNER in epirubicin-induced apoptosis. To further confirm the protective role of DNER against epirubicin-induced apoptosis, we measured cell viability and apoptosis after overexpressing DNER. The CCK-8 assay showed that DNER overexpression could partially rescue the inhibition of cell proliferation induced by epirubicin (Fig. 7h). Unlike the effects of DNER knockdown determined by FACS and western blotting, DNER overexpression significantly antagonized epirubicin-induced apoptosis in BC cells (Fig. 7i, j). Taken together, these results demonstrate that DNER protects BC cells from epirubicin-induced apoptosis.

### Regulation of the Wnt/β-catenin pathway by DNER is involved in epirubicin-induced apoptosis

**Fig. 8:**
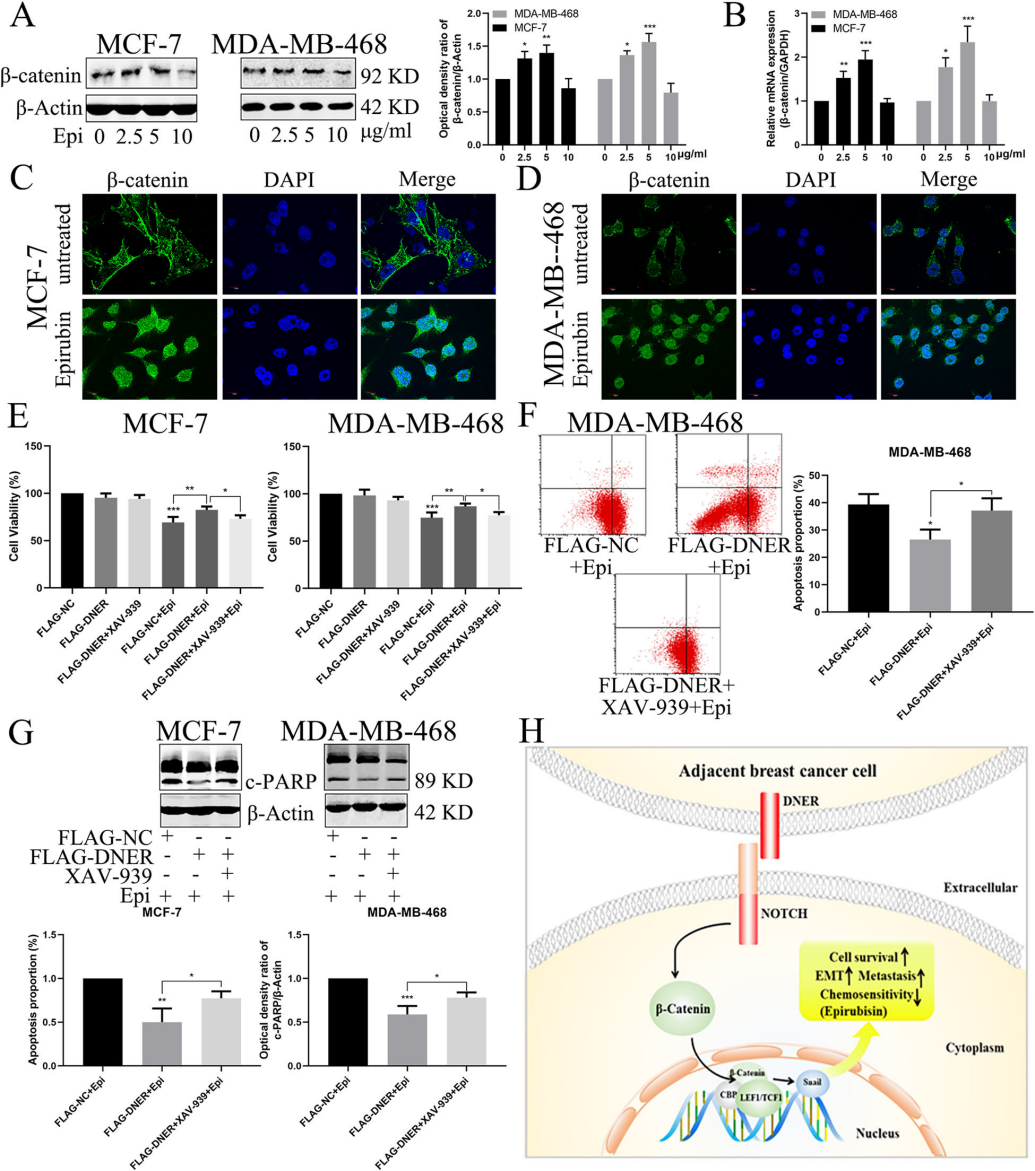
Regulation of the Wnt/β-catenin pathway by DNER is involved in epirubicin-induced apoptosis. **a** β-catenin was analyzed by western blotting in BC cells treated with different concentrations of epirubicin. Right: quantitative analysis of the optical density ratio of β-catenin compared with β-actin are shown. **b** Expression of epirubicin-induced DNER detected by real-time RT-PCR. **c**, **d** Distribution of β-catenin in BC cells treated with epirubicin that were analyzed with confocal microscopy. β-catenin is stained green, and the nucleus is stained blue. Scale bar = 10 μm. **e** Cells were transfected with FLAG-NC or FLAG-DNER, and then treated with XAV-939 for 24 h before epirubicin treatment. Cell proliferation was detected by CCK-8. **f** Analysis of apoptosis with FACS in MDA-MB-468 cells treated as described above. Right: Quantitative analysis of apoptosis ratio. **g** The expression of PARP was detected by western blotting in BC cells treated as described above. Right: quantitative analysis of the optical density ratio of c-PARP compared with β-actin are shown. The values are the mean ± SD from three independent experiments. *p < 0.05, **p < 0.01, ***p < 0.001 vs the corresponding group. **h** Schematic model for DNER-induced biological function of BC cells.

We have previously shown that DNER can regulate the Wnt/β-catenin signalling pathway. To determine whether β-catenin is involved in the epirubicin-induced apoptosis of BC cells, two BC cell lines were treated with different concentrations of epirubicin. As shown in Fig. 8a, the protein levels of β-catenin in BC cells were increased in an epirubicin concentration-dependent manner. Furthermore, upregulation of β-catenin induced by epirubicin occurred at the transcriptional level (Fig. 8b). More importantly, β-catenin was transferred into the nucleus after epirubicin treatment (Fig. 8c, d). We next examined cell growth and apoptosis in DNER-overexpressing cells in the absence and presence of XAV-939. A CCK-8 assay showed that the treatment of DNER-overexpressing cells with XAV-939 augmented the epirubicin-induced inhibition of cell proliferation compared with that when DNER was overexpressed alone (Fig. 8e). In addition, FACS revealed that the rate of epirubicin-induced apoptosis in DNER-overexpressing MDA-MB-468 cells was significantly elevated after XAV-939 treatment (Fig. 8f). Furthermore, changes in the levels of apoptosis-related proteins indicated the same outcome as FACS (Fig. 8g). These results showed that β-catenin amplified the rate of epirubicin-induced apoptosis, which was reversed by DNER; therefore, we conclude that DNER inhibits epirubicin-induced cell apoptosis via the Wnt/β-catenin signalling pathway (Fig. 8h).

## Discussion

In our study, we have provided comprehensive results on the role of DNER in regulating BC EMT and chemosensitivity. We found that the expression level of DNER in BC tissue, especially TNBC tissue, is obviously higher than that in adjacent tissue and that high DNER expression is associated with poor prognosis in BC and TNBC patients. Furthermore, DNER not only clearly enhanced cell viability, migration, invasion and EMT in vitro but also significantly augmented the tumorigenic and metastatic ability in vivo. In addition, we demonstrated that Wnt/β-catenin is essential for DNER-mediated cell growth, migration, invasion and EMT and that patients with high levels of DNER and β-catenin exhibit worse survival. Finally, DNER protects BC cells from epirubicin-induced apoptosis via the Wnt/β-catenin pathway. Therefore, DNER functions as an oncogene in BC and may be a prognostic factor and therapeutic target for BC.

DNER, atypical ligand of the Notch1 pathway, was initially found to be highly expressed in Purkinje neurons and plays a key role in cerebellar development^25,26^. Furthermore, the Notch pathway is involved in carcinogenesis and promotes the proliferation and malignant progression of various tumours^27–29^. Until now, the role of DNER in cancer has remained controversial, and the function of DNER may vary according to cell type. Several studies have shown that knockdown of DNER by siRNA or shRNA inhibits cancer cell proliferation, migration and invasion in vitro and reduces the size of some solid tumours in vivo^16,17,30^. However, in glioblastoma, the specific gene product induced by DNER-mediated histone deacetylase (HDAC) inhibition inhibits the growth of GBM-derived neurospheres, induces differentiation in vitro and in vivo and inhibits tumour xenograft growth^14^. Our results demonstrated that DNER remarkedly promoted the cell growth, migration and invasion of BC cell lines in vitro and augmented the tumorigenic and metastatic ability in vivo. These inconsistent findings suggest that the role of DNER is tissue- or cell-specific or that HDAC inhibitors inhibit the proliferation of glioblastoma by inhibiting the expression of DNER, thereby protecting the body. Furthermore, DNER could reduce the RFS in BC patients, especially TNBC patients, suggesting DNER as a prognostic indicator in BC, especially TNBC.

A growing body of research suggests that the distant metastasis of tumours is caused by EMT of tumour cells, which promotes cancer cell metastasis^10,31^. In addition, DNER acts as a ligand for Notch1, activating the Notch signalling pathway^11,15^, and the knockdown of Notch1 expression can inhibit the migration and invasion of nasopharyngeal carcinoma cells by reversing EMT^32^. In our study, the knockdown of DNER significantly increased the expression of epithelial markers and inhibited the expression of interstitial markers, while DNER overexpression had the opposite effect. Furthermore, overexpression of DNER in DNER knockdown cells reversed the EMT phenotype. These results revealed that DNER promotes BC progression through regulating cell EMT.

The canonical Wnt/β-catenin pathway plays a critical role in the apoptosis, proliferation and metastasis of cancer cells^10,33^. Our research demonstrated that DNER observably activates the Wnt/β-catenin pathway to regulate the proliferation, migration, invasion and EMT of BC cells. This finding was verified by nuclear and cytoplasmic separation experiments and downstream target gene detection with qRT-PCR after DNER knockdown. Furthermore, CHIR 99021 and XAV-939 reversed change to cellular biological functions and the EMT phenotype after DNER knockdown and overexpression, respectively. In addition, we demonstrated a positive correlation between DNER and catenin. Furthermore, BC patients with combined high expression levels of DNER and β-catenin exhibited worse survival. Hence, Wnt/β-catenin is not only regulated by DNER but also participates in the prognosis of BC patients together with DNER.

The effect of β-catenin and EMT on cell apoptosis has been reported^34,35^, but the role of DNER in epirubicin-induced apoptosis has not been studied. DNER and β-catenin were expressed in an epirubicin concentration-dependent manner. Knockdown of DNER significantly increased the epirubicin-induced inhibition of cell proliferation and apoptosis, while overexpression of DNER had the opposite effect. More interestingly, the extent to which epirubicin inhibited proliferation and cell apoptosis in DNER-overexpressing cells was dramatically enhanced after XAV-939 treatment. These results indicated that DNER protects BC cells from epirubicin-induced growth inhibition and apoptosis via the Wnt/β-catenin pathway.

In conclusion, we have demonstrated the crucial functional role of DNER in EMT and apoptosis. Our study revealed that DNER is highly expressed and associated with poor survival in BC patients, especially TNBC patients, as shown for the first time. In addition, DNER promotes BC cell invasion, EMT and the rate of apoptosis by regulating the Wnt/β-catenin pathway. Therefore, although further research is needed, our study provides comprehensive evidence demonstrating the value of DNER in EMT and apoptosis and suggests DNER as a worthwhile therapeutic target for BC.

## Supplementary information

Supplemental Figure 1

Supplemental Figure 2

Supplemental Figure 3

Supplementary Figure Legend

supplemental Table 1

supplemental Table 2
