# Supplementary figures and images for: DNER promotes epithelial–mesenchymal transition and prevents chemosensitivity through the Wnt/β-catenin pathway in breast cancer
Source: Cell Death Dis. 2020 Aug 18;11(8):642. doi: 10.1038/s41419-020-02903-1 (PMC7434780; doi:10.1038/s41419-020-02903-1)

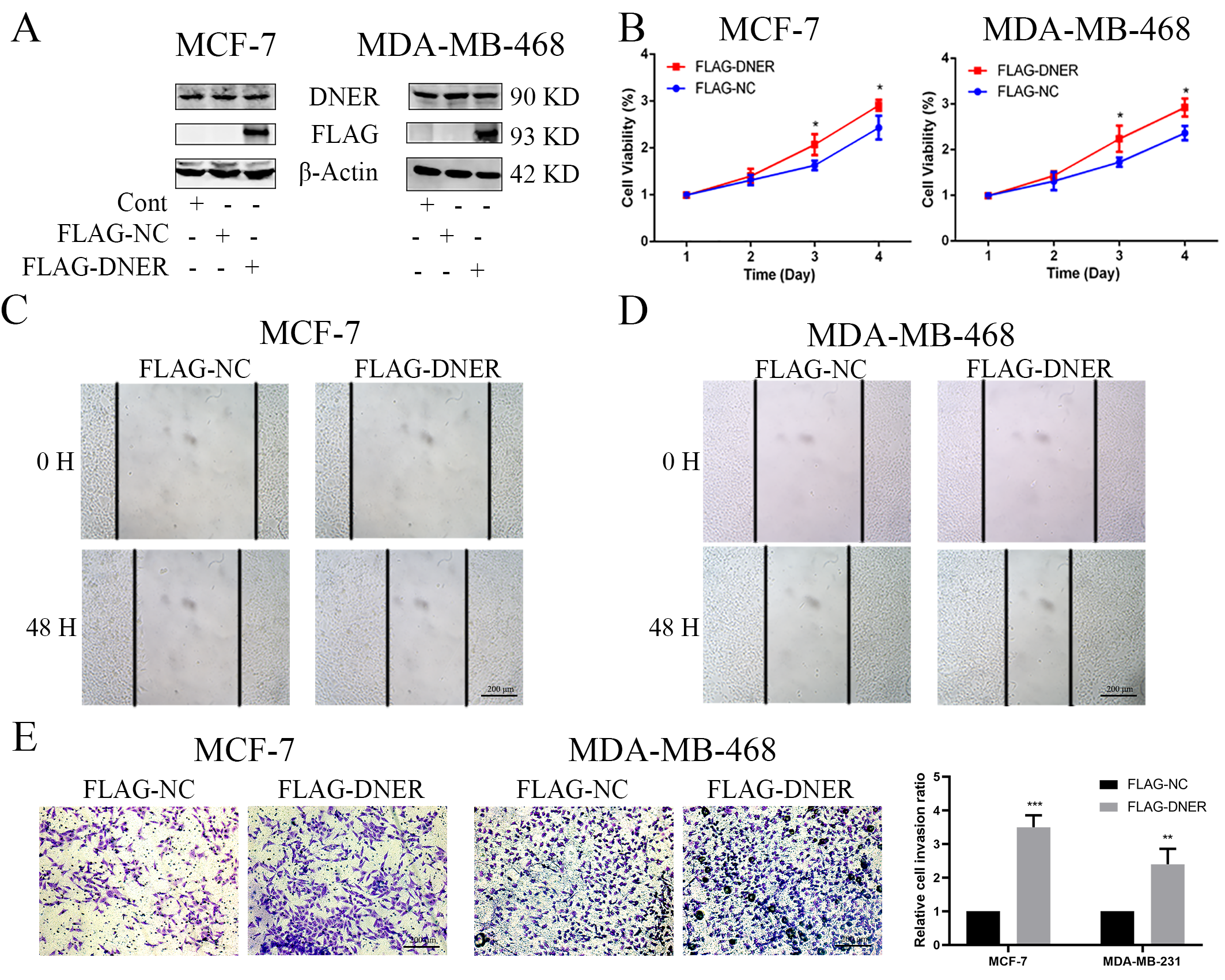

Supplement: Supplementary file 1 — Supplemental Figure 1 [file 41419_2020_2903_MOESM1_ESM.tif]

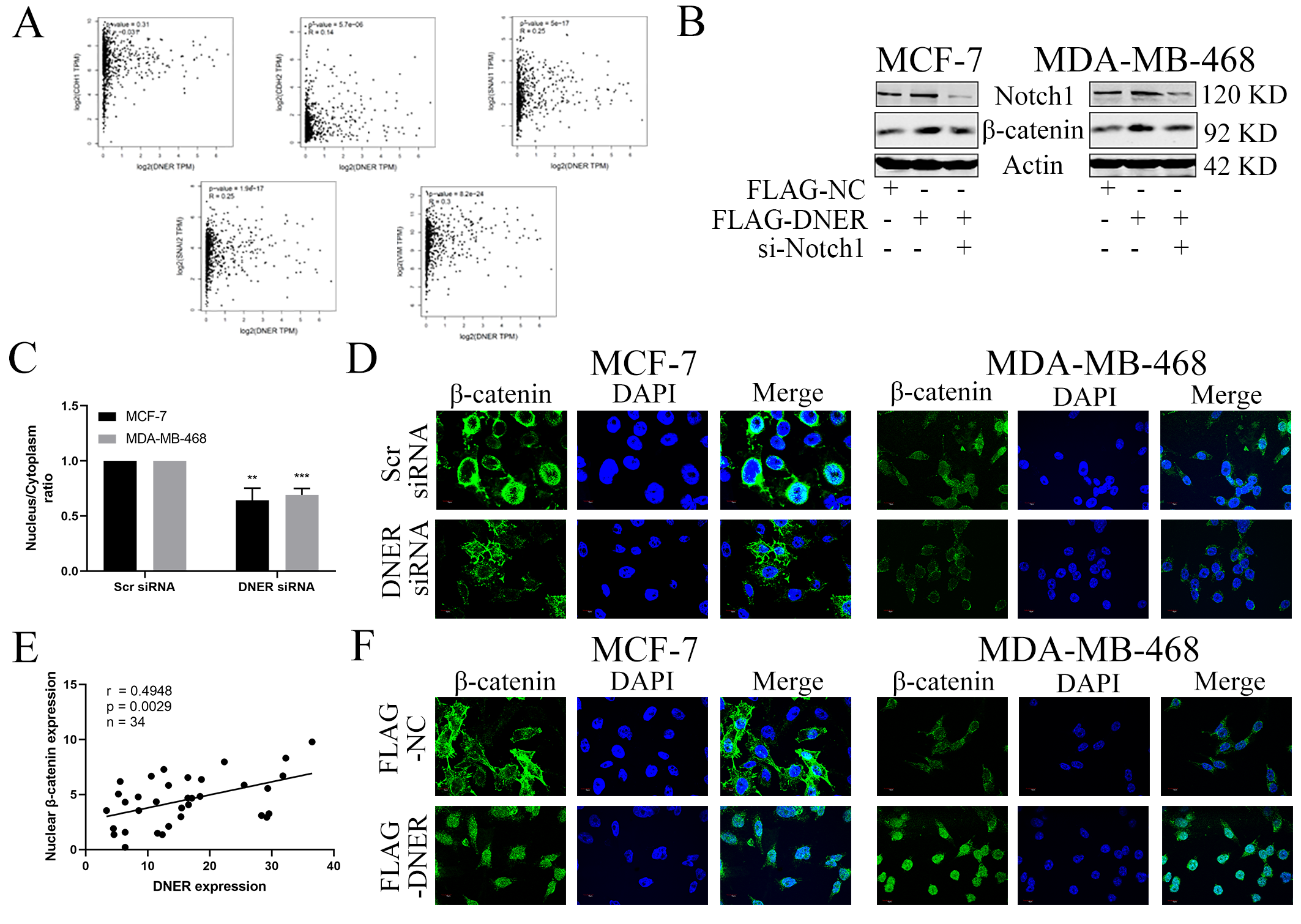

Supplement: Supplementary file 2 — Supplemental Figure 2 [file 41419_2020_2903_MOESM2_ESM.tif]

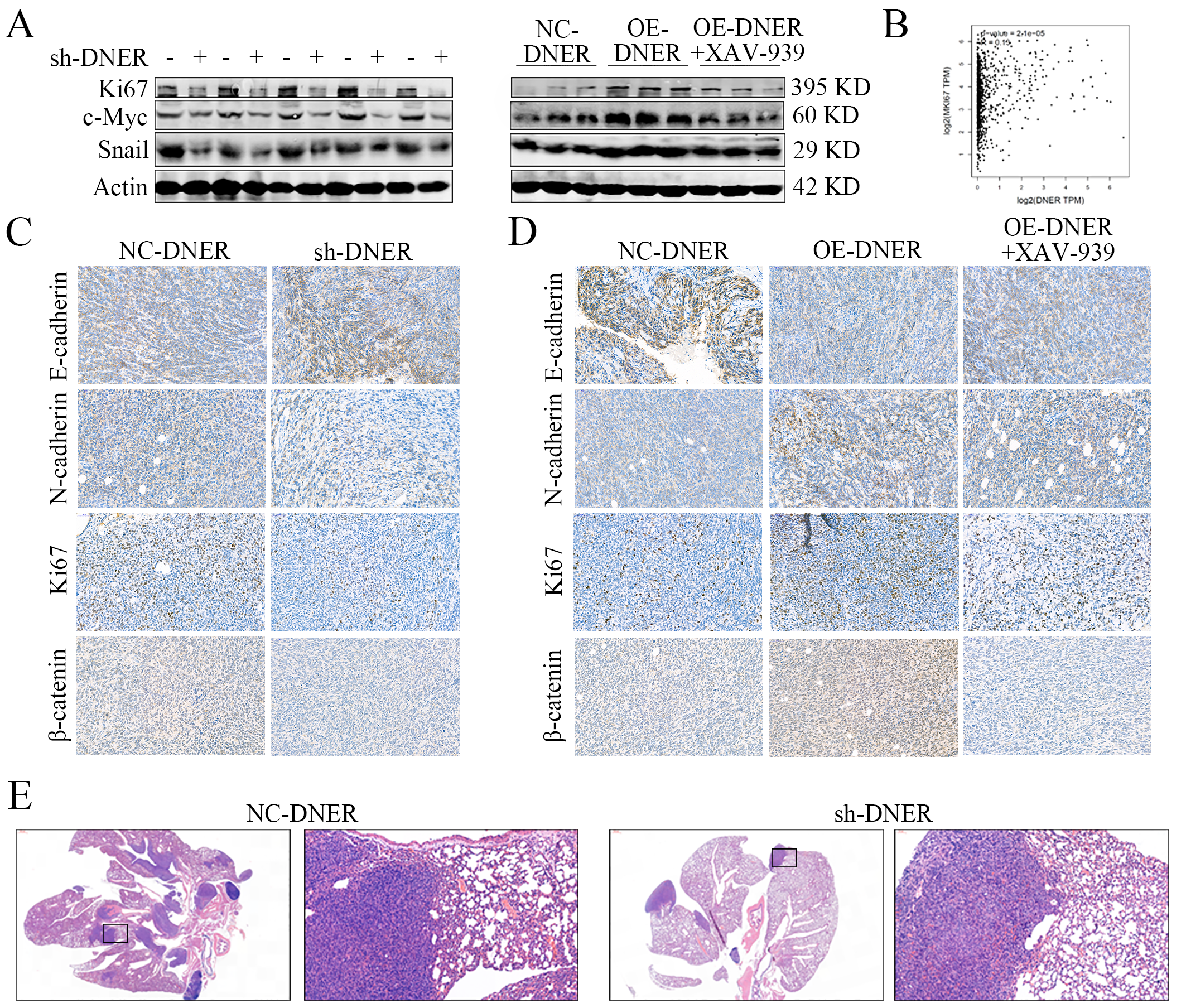

Supplement: Supplementary file 3 — Supplemental Figure 3 [file 41419_2020_2903_MOESM3_ESM.tif]
