# Supplementary material for: DNER promotes epithelial–mesenchymal transition and prevents chemosensitivity through the Wnt/β-catenin pathway in breast cancer
Source: Cell Death Dis. 2020 Aug 18;11(8):642. doi: 10.1038/s41419-020-02903-1 (PMC7434780; doi:10.1038/s41419-020-02903-1)
Supplement: Supplementary file 4 — Supplementary Figure Legend [file 41419_2020_2903_MOESM4_ESM.docx]

**Supplementary Figure Legends**

**Supplementary Figure 1. Overexpression of DNER promotes cell proliferation and metastasis of BC cells.** (A) The overexpression efficiency of DNER in MCF-7 and MDA-MB-468 cells. (B) Cell proliferation was detected by CCK-8 assay after DNER overexpression in BC cells. (C-D) The capacity of migration by overexpression of DNER was measured with wound healing assay. (E) Invasion ratio was detected by Transwell assay in BC cells with DNER overexpression. Right: Quantitative analysis of invasion ratio was shown. The values are the mean±SD from three independent experiments. ^*^p<0.05, ^**^p<0.01, ^***^p<0.001 vs the control group.

**Supplementary Figure 2.** **DNER correlated with β-catenin and EMT related markers, respectively.** (A) Correlation analyses of mRNA expression levels between DNER with E-cadherin, N-cadherin, Snail, Slug and Vimentin from TCGA database, respectively. (B) Notch1 was knocked down in DNER-overexpressing cells, and then Western blotting detected the expression of Notch1 and β-catenin. (C) Quantitative analysis of Nuclear/Cytoplasm ratio after DNER knockdown in BC cells. (D and F) Distribution of β-catenin after DNER knockdown or DNER overexpression in BC cells that were analyzed with confocal microscopy. β-catenin is stained green, and the nucleus is stained blue. Scale bar=10 μm. (E) Correlation analyses of protein expression levels between DNER and nuclear β-catenin.

**Supplementary Figure 3. Detection of related protein levels in xenograft.** (A) The expression of Ki67, c-Myc and Snail in xenograft tissue by Western blotting. (B) Correlation analyses of mRNA expression levels between DNER and Ki67 from TCGA database. (C-D) The expression of E-cadherin, N-cadherin, Ki67 and β-catenin in xenograft tissue by IHC. (E) Histological analyses of lung metastatic tumors by haematoxylin and eosin (H&E) staining.
