# Supplementary material for: DNER promotes epithelial–mesenchymal transition and prevents chemosensitivity through the Wnt/β-catenin pathway in breast cancer
Source: Cell Death Dis. 2020 Aug 18;11(8):642. doi: 10.1038/s41419-020-02903-1 (PMC7434780; doi:10.1038/s41419-020-02903-1)
Supplement: Supplementary file 5 — supplemental Table 1 [file 41419_2020_2903_MOESM5_ESM.docx]

**Sup.Tab.1 Primary antibodies and secondary antibodies for Western blotting**

| Antibody | Concentration | Article number | Company |
| --- | --- | --- | --- |
| DNER | 1:500 | sc-100305 | Santa Cruz |
| E-cadherin | 1:200 | sc-7870 | Santa Cruz |
| N-cadherin | 1：1000 | 13116 | Cell Signaling Technology |
| β-catenin | 1：1000 | 8480 | Cell Signaling Technology |
| NOTCH1 | 1：1000 | 3608 | Cell Signaling Technology |
| p-GSK3β | 1：1000 | 9323 | Cell Signaling Technology |
| GSK3β | 1：1000 | 9315 | Cell Signaling Technology |
| Snail | 1：1000 | 3879 | Cell Signaling Technology |
| Vimentin | 1：1000 | 5741 | Cell Signaling Technology |
| Lamin B1 | 1：1000 | 13435 | Cell Signaling Technology |
| FLAG  c-Myc  Ki67 | 1：5000  1:1000  1:200 | F1804  18583  sc-23900 | Sigma  Cell Signaling Technology  Santa Cruz |
| β-Actin | 1：10000 | A5441 | Sigma |
