# Supplementary material for: DNER promotes epithelial–mesenchymal transition and prevents chemosensitivity through the Wnt/β-catenin pathway in breast cancer
Source: Cell Death Dis. 2020 Aug 18;11(8):642. doi: 10.1038/s41419-020-02903-1 (PMC7434780; doi:10.1038/s41419-020-02903-1)
Supplement: Supplementary file 6 — supplemental Table 2 [file 41419_2020_2903_MOESM6_ESM.docx]

| Gene | Sequence |
| --- | --- |
| DNER | F: CAGGGACCTCGTTAATGGCT  R: CCGTTCAGACAGCTGACGTT |
| Survivin | F: CCACCGCATCTCTACATTCAA  R: CAAGTCTGGCTCGTTCTCAGG |
| c-Myc | F: GTCAAGAGGCGAACACACAAC  R: TTGGACGGACAGGATGTATGC |
| LEF1  β-catenin | F: TGGATCTCTTTCTCCACCCA  R: TGGATCTCTTTCTCCACCCA  F: TGGTGCCCAGGGAGAACCCC  R: TGTCACCTGGAGGCAGCCCA |
| GAPDH | F: AGAAGGCTGGGGCTCATTTG  R: AGGGGCCATCCACAGTCTTC |

**Sup.Tab.2 The sequence of primers for qPCR**
